# The physicochemical, microbiological, and organoleptic properties and antioxidant activities of cream cheeses fortified with dried curry leaves (*Murraya koenigii* L.) powder

**DOI:** 10.1002/fsn3.2551

**Published:** 2021-08-30

**Authors:** Dilshani Weragama, Viraj Weerasingha, Lakmini Jayasumana, Jayantha Adikari, Janak K. Vidanarachchi, Hasitha Priyashantha

**Affiliations:** ^1^ Faculty of Agriculture Department of Animal & Food Sciences Rajarata University of Sri Lanka Anuradhapura Sri Lanka; ^2^ Faculty of Agriculture Department of Animal Science University Peradeniya Peradeniya Sri Lanka; ^3^ Department of Molecular Sciences Swedish University of Agricultural Sciences Uppsala Sweden

**Keywords:** antioxidants, cream cheese, curry leaves, natural plant ingredients, total phenolic content

## Abstract

We aimed to investigate the effects of dried curry leaves powder (CLP) incorporation on physicochemical, microbiological, antioxidant, and sensory properties of cream cheeses. Varying levels of CLP infusions (i.e., T1: 0% [control], T2: 0.15%, T3: 0.2%, and T4: 0.25%; w/w%) were stored for 10 days at 4°C. Antioxidant properties were evaluated using total phenolic content, 1,1‐diphenyl‐2‐picryl‐hydrazyl (DPPH) radical scavenging ability, and ferric reducing antioxidant power using in vitro assays. Total antioxidant capacity significantly (*p* < .05) increased with the increasing levels of CLP. Physicochemical and microbiological qualities were not significantly affected by the addition of CLP, indicating the suitability of using CLP without compromising the quality of cream cheese. Organoleptic properties were affected with CLP addition, where T3 had the highest scores for color, aroma, flavor, texture, and overall acceptability. The principal component analysis provides the holistic approach of studying the variation associated with cream cheeses and the overall relationship among studied parameters. This provides strong references for novel dairy products added with antioxidant‐rich *Murraya koenigii* L. powder. The study also has merits to promote scientific knowledge concerning, and how the incorporation would influence the physicochemical, organoleptic, and microbiological properties of cream cheese to deliver the value‐added or diversified product to emerging consumers.

## Graphical abstract

This study shows inclusion of dried curry leaves powder into cream cheese increased antimicrobial and antioxidative attributes with enhanced organoleptic properties, while physicochemical attributes remained unaffected.

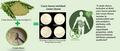

## 1 INTRODUCTION

The demand for value‐added dairy products is ever‐growing, where consumers are seeking various functional and bioactive properties. As an attempt, diversification of cream cheese with several herbs and spices has been practiced. Cream cheese is a soft and white‐colored cheese with a subtle and sweet flavor. Often, it is used as a spread (e.g., for bread, bagels, and crackers) and a dip (e.g., for potato chips) and used as an ingredient in many food applications (Sainani et al., 2004). Cream cheese is mainly acidified with mesophilic lactic acid starter cultures consist of *Lactococcus* spp. and *Leuconostoc* spp. while rennet is commonly used as the coagulant (Phadungath, 2005). According to the U.S Food and Drug Administration (2019), cream cheese must have a minimum milk fat content of 33% and maximum moisture content of 55% by weight. Dairy products that contain high amounts of polyunsaturated fatty acids are easily oxidized by molecular oxygen as the storage period lengthens. It causes unfavorable changes such as loss of flavor, loss of color, and accumulation of compounds that are detrimental to the health of the consumers (Gad & Sayd, 2015). At present, dairy producers tend to add various types of synthetic antioxidants (i.e., propyl gallate, butylated hydroxyanisole, and butylated hydroxytoluene, etc.) to prolong the shelf life of various processed dairy products (Safriani et al., 2011). There is a trend among modern consumers to purchase food commodities supplemented with natural compounds other than synthetic substances (Kitts & Tomiuk, 2013), thus creating a need for developing products with natural compounds by replacing the use of artificial additives. Natural antioxidants such as ascorbic acids, lycopene, and tocopherols and some flavonoids are preferred to be added into foods and they strengthen the activity of endogenous antioxidant systems thus providing elevated protection against antioxidant stress (Valenzuela et al., 2003). Substances of plant origin have been traditionally used in cheese making, either to add aromatic properties or to provide technological auxiliaries such as milk‐clotting enzymes or cheese wrappers. Some of these plants (i.e., rosemary, thyme, olive essential oil, etc.) are known to have antimicrobial and antioxidant properties and also act as natural preservatives for raw milk and milk‐derived products (Dupas et al., 2020).

*Murraya koenigii* L. which belongs to Rutaceae family is commonly identified as curry leaves. It has wide popularity in Asian countries for its aroma and medicinal properties (Bhandari, 2012; Handral et al., 2012). The leaves of *Murraya koenigii* L. are a rich source of protein, dietary fiber, iron, calcium, β‐carotene, and antioxidants (Drisya et al., 2015). A study by Khan et al. (1997) showed that *Murraya koenigii* L. and *Brassica juncea* spices alter the peroxidation level to a beneficial extent in rats fed with a high‐fat diet. Phenolic‐rich benzene fraction of *Murraya koenigii* L. has exhibited promising and broad‐spectrum antioxidant and antimutagenic properties in previous studies (Sasidharan & Menon, 2010; Zahin et al., 2013). Given the potentials of antioxidant and antimicrobial activities of *Murraya koenigii* L., it is not known how exactly those properties are exhibiting when *Murraya koenigii* L. is incorporated into dairy matrixes. Therefore, the objective of the current study was to evaluate the physicochemical, microbiological, sensory quality, and antioxidant capacity of fresh cream cheese incorporated with dried *Murraya koenigii* L. powder to assess the potentials in developing a functional food.

## 2 MATERIALS AND METHODS

### 2.1 Preparation of dried curry leaves powder

Fresh curry leaves were collected from the Matale area, Sri Lanka. Plants were botanically verified as *Murraya koenigii* (L.) Spreng by National Herbarium, Department of National Botanical Gardens, Peradeniya, Sri Lanka. The dried curry leaf powder was prepared according to the following method (Mishra et al., 2012) with few modifications. In brief, curry leaves were soaked in 1% saline solution (NaCl) for 10 min and subsequently washed twice with distilled water. Cleaned leaves were dried under direct sunlight for 5–6 h. Dried curry leaves were powdered using Super Mixer Grinder (National MX‐119N) and sieved through a 100‐µm sieve and stored in airtight containers until further use.

### 2.2 Preparation of cream cheese

Cream cheese was prepared in triplicate according to a modified method (Enwa et al., 2013). Fresh cow milk was strained and standardized for the final fat content of 3.5%. Milk base was pasteurized at 63–68°C for 30 min followed by cooling down to 30–32°C. Subsequently, 0.075% w/v mesophilic starter culture (CHN‐22, Chr. Hansen) with the composition of *Lactococcus lactis subsp. cremoris, Leuconostoc, Lactococcus lactis subsp. lactis, and Lactococcus lactis subsp. lactis biovar diacetylactis* was added along with 0.05% w/v fermentation‐produced bovine calf chymosin (chymosin 100%, 650 International Milk‐Clotting Units per gram (IMCU/g); CHY‐MAX). The milk base was then incubated for 40–45 min until milk curd was formed. Curd was cut with a pasteurized spatula and whey was left to drain through cheese clothes for 1 h. Salt (2% w/w maximum) and different levels of CLP were added making four types of cream cheeses (i.e., T1 = 0% CLP (control), T2 = 0.15% CLP, T3 = 0.2% CLP, and T4 = 0.25% CLP, w/w) and mixed well using a food processor (Jaipan Family Mate). Processed cream cheese samples were stored in sterilized airtight containers at 4°C until further analyses.

To determine the appropriate levels of CLP incorporation, a preliminary sensory evaluation was performed using 30 untrained panelists with a 5‐point hedonic scale. Initially, we tested the possibilities of including four levels of CLP inclusions (i.e., 0.2%, 0.4%, 0.6%, and 0.8% (w/w) into cream cheese and determined that the 0.2% (w/w) CLP inclusion level was best preferred by the panelists (data not shown). Because of better organoleptic quality as revealed from the preliminary sensory evaluation, we opted for the 0.2% (w/w) level of inclusion for further investigations and changed the level of inclusion slightly around this level to find the best possible level of incorporation in the present study.

### 2.3 Physicochemical properties

#### 2.3.1 pH and titratable acidity

Cream cheese pH was measured using a pH meter (SensION) during the storage period of 10 days. Briefly, a slurry was created with cheese: distilled water in a 1:1 ratio at 20°C and the pH of the suspension was measured with the standard laboratory electrode‐based pH meter in triplicates (Burdikova et al., 2015). Titratable acidity was measured according to the method described by with few modifications suggested by Jayarathna et al. (2020). In brief, the filtrates of cream cheese were titrated against 0.1% NaOH in the presence of phenolphthalein indicator until a persistent pale pink color was observed. Titratable acidity was calculated based on the lactic acid percentage using Equation (1).

(1) $$\mathrm{Lactic acid}\left(\%\right)=\frac{9\;\left(\text{The volume of}\;0.1\text{N NaOH}\times \text{Normality of standard NaOH}\right)}{\text{Volume in mL of yogurt taken for the test}}$$

#### 2.3.2 Syneresis

Five grams (5 g) of cream cheese was measured into an enclosed tube and centrifuged for 30 min at 986 × *g*, at 20°C. The yield of whey was calculated according to Equation (2) as the percentage of weight drained from each sample after centrifugation (Cadavid et al., 2019; Jayarathna et al., 2020) using the averages of triplicates.

(2) $$\text{Syneresis}\left(\%\right)=\frac{\text{Weight of whey}}{\text{weight of sample}}\times 100\%$$

#### 2.3.3 Moisture, total solids, and crude fat content

Moisture and total solids content of cream cheese were determined according to the methods described by Bradley (2010) and Regu et al. (2016), respectively, on triplicated cheeses. Crude fat content was determined by the Soxhlet extraction method as described by Min and Ellefson (2010), with certain modifications such as extracting samples for 10 h with petroleum ether.

### 2.4 Microbiological analysis

Total viable aerobic counts, coliform counts, and yeast and mold counts of cream cheese were evaluated throughout the entire storage period at day 1, day 5, and day 10 based on triplicates. A serial dilution of cream cheese was prepared using 0.1% peptone (CDH) solution, and diluted samples were plated using the spread plate technique. Plate count agar (Biolab) and coliform count agar (Oxoid CM0007, MacConkey Agar) were used for the enumeration of total bacterial load and *E. coli* and other coliform bacteria, respectively, at 37°C for 24 h. For the enumeration of yeast and mold counts, potato dextrose agar (Biolab) was used while plates were incubated at 30°C for 72 h.

### 2.5 Antioxidant assays

#### 2.5.1 Sample preparation

Methanol extractions of cream cheese samples were prepared in triplicates (Parekh et al., 2006) with the following modifications. Cream cheese samples (5 g) were extracted with 50 ml of 80% methanol, keeping a solvent to cream cheese ratio of 1:10. The upper phase was filtered with Whatman No. 1 filter paper and kept at 4°C in airtight containers until further analyses.

#### 2.5.2 Determination of total phenolic content of cream cheese samples

The Folin Ciocalteu's colorimetric method was used to determine the total phenolic content (TPC) of the CLP‐incorporated cream cheese samples as originally described by Singleton and Rossi (1965) and Parekh et al. (2006) as follows: Methanol extract (0.5 ml) was transferred into a 10‐ml volumetric flask mixed containing 1.5 ml of freshly diluted (1:1 v/v) Folin Ciocalteu's reagent and 1.0 ml of 10% sodium carbonate solution. It was volumed up with distilled water. The samples were subjected to stand at ambient temperature (27°C) for 2 h for color development (dark blue). The absorbance of the clear supernatant was measured at 765 nm using a spectrophotometer (Agilent Technologies: Cary 60 UV‐Vis). Gallic acid was used to prepare the standard series. The results were expressed as milligrams of gallic acid equivalents per hundred grams of the sample (mg GAE/100 g of cream cheese).

#### 2.5.3 Determination of 2,2‐diphenyl‐1‐picryl‐hydrazyl radical scavenging ability

The DPPH (2,2‐diphenyl‐l‐picryl‐hydrazyl) radical scavenging ability of the extracts was evaluated according to the method described by Brand‐Williams et al. (1995). Sample extracts were prepared with concentrations of 0.025, 0.050, 0.075, and 0.1 g/ml. The diluted extract (2 ml) was taken into a test tube, and it was mixed with another 2 ml of DPPH (3.9 mg of DPPH in 100 ml of 99.8% methanol), mixed thoroughly, and kept in dark for 30 min. The absorbance was measured at 515 nm along with a blank sample (99.8% methanol) via a UV‐visible spectrophotometer (Agilent Technologies). The percentage of radical scavenging ability was calculated according to Equation (3).

(3) $$Radicalscavengingability=[\left(Acontrol‐Asample\right)/Acontrol]\;\times \;100\%$$

where

A _control_ = Absorbance of the blank and A _sample_ = Absorbance of the sample.

The IC_50_ value was calculated to determine AEAC (ascorbic acid equivalent antioxidant capacity) and it was derived from the scavenged DPPH percentage versus concentration plot (not shown). The results also were expressed as AEAC using Equation (4) (Lim et al., 2007).

(4) $$AEAC=[IC_{50}\left(\mathrm{AA}\right)/IC_{50}\left(\mathrm{sample}\right)\times 10^{5}$$

where

IC_50_ (AA) = IC_50_ value of ascorbic acid and IC_50_ (sample) = IC_50_ value of the extract sample.

#### 2.5.4 Determination of ferric reducing antioxidant power assay

The FRAP assay employs the conversion of ferric to ferrous ion reduction at low pH, causing to form an intense blue colored ferrous‐tripyridyltriazine (Fe^II^‐TPTZ) from ferric‐tripyridyltriazine (Fe^III^‐TPTZ) complex. The FRAP values are determined by comparing the absorbance change at 593 nm in test reaction mixtures, with those containing ferrous ions in known concentrations (Lim et al., 2007). In the present study, the FRAP assay was performed according to the following method (Benzie & Strain, 1996).

Methanolic cream cheese extract (0.5 ml) was mixed with 9 ml of distilled water and obtained 1 ml of extract to mix with 6.0 ml of FRAP reagent. The samples were vortexed until mixed, and they were incubated for 30 min at 37°C until the color development was completed. The absorption was measured at 593 nm using the spectrophotometer (Agilent Technologies: Cary 60 UV‐Vis). Regression equation design for the standards was used to calculate the FRAP values (mmol of Fe [II] equivalents per 1 g of sample).

### 2.6 Sensory evaluation

Sensory analysis of CLP‐incorporated cream cheese samples was carried out using 30 untrained panelists (aged 20–45 years) according to a randomized design using the triplicated cheese production. Each panelist scored the cream cheeses (T1 to T4) according to a 5‐point hedonic scale (1–5, from strongly disliked to strongly liked, respectively) using a simplified and structured sensory evaluation ballet at 25°C. For the evaluation, panelists considered the flavor, color, texture, aroma, and overall acceptability of the cream cheese samples. The same score was not given for two or more samples.

### 2.7 Statistical analysis

Physicochemical and microbiological attributes were analyzed using one‐way ANOVA of minitab software (Minitab^®^ 17.1.0) with a 95% confidence interval (significance at the *p* < .05). The Tukey test was used for mean separation in physicochemical and microbiological attributes while the Friedman test was used for sensory evaluation. Antioxidant data were analyzed by the completely randomized design of ANOVA of sas program (version 9.0, SAS Inc) and mean separation among the treatments was performed by least‐square difference (LSD) method to determine the statistical difference among them at the significant level of *p* < .05. Principal component analysis (PCA) was used in multivariate analyses of all studied observations, using the software simca 16.0 (Sartorius Stedim Data Analytics AB). The variables were preprocessed with UV centering. PCA score scatter plots were developed for assessing similarities and groupings of studied parameters, while PCA loading scatter plots were used to interpret the score to scatter plots to display similarities or differences among all variables.

## 3 RESULTS AND DISCUSSION

### 3.1 Changes in pH, titratable acidity, syneresis of cream cheese during the storage period

**FIGURE 1:**
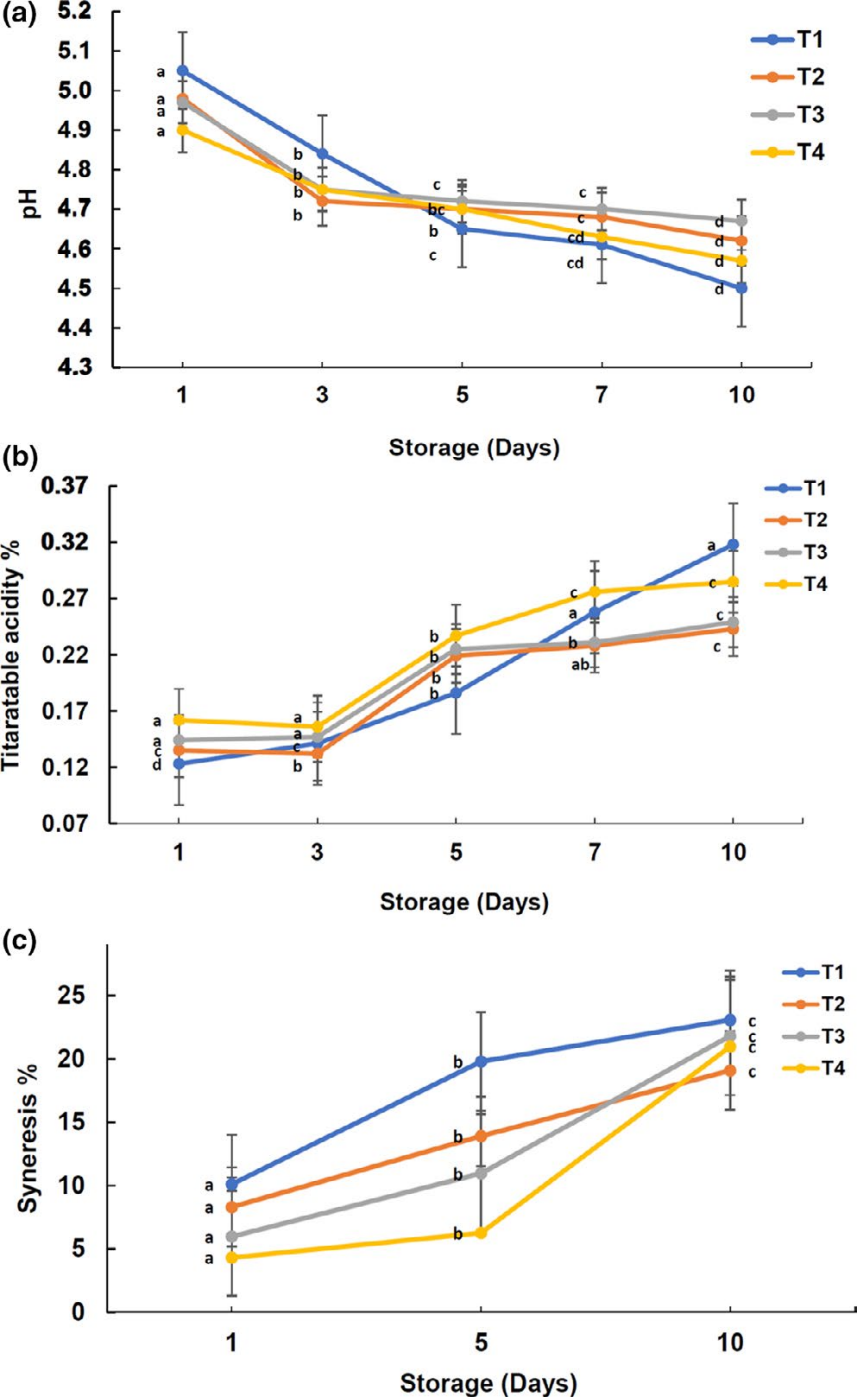
Changes of (a) pH and (b) titratable acidity (c) syneresis of cream cheese during storage at 4°C; standard error bar is shown; means with different lowercase letters are significantly different (*p* < .05) between each treatment of cream cheese, for a particular day of storage; T1, T2, T3, and T4 contain varying levels of dried CLP w/v = 0% (control), 0.15%, 0.2%, and 0.25%, respectively

Since the paper is in large part about processing and preservation of cream cheese with plant materials, physicochemical properties were evaluated during the stipulated shelf life. Treatment (incorporation of CLP) did not significantly (*p* > .05) influence the pH, titratable acidity (TA), or syneresis (Figure 1). During the storage period, however, there was a gradual decrease (*p* < .05) in pH (Figure 1a) from 5.05 ± 07 to 4.57 ± 0.04, while increase (*p* < .05) in titratable acidity (TA; Figure 1b) from 0.141 ± 0.02 to 0.285 ± 0.01 and syneresis from 4.31 ± 0.04 to 23.01 ± 0.04 (Figure 1c) for all the treatments. In fact, we observed an interactive effect on cream cheese pH with the storage time, as the initial pH was higher than the CLP‐treated cream cheese, and at the end of storage, pH was lower than the rest of the samples. This perhaps, the addition of CLP may buffer the pH, and therefore, drop in pH is seemingly higher for the control. Similarly, Olmedo et al. (2013) observed that the untreated cream cheese showed lower pH values than the samples treated with plant‐based extracts (Oregano and rosemary essential oil) during storage. The gradual increase of TA in cream cheese samples might be an effect of the fermentative deterioration of dairy products over the storage according to Galán et al. (2008), who showed a development of sour taste of cheese made with sheep milk, due to an increase in residual lactose fermentation, because of increased activity of lactic acid bacteria. Likewise, in the present study, the total viable counts increased during the storage (Table 2), while pH decreased and thus the TA increased. Our results are in agreement with Perveen et al. (2011), who observed gradual increases in titratable acidity with the storage of cream cheese at 4 ± 1°C. The lower TA in CLP‐treated cream cheese samples in contrast to the control sample demonstrates that CLP inhibited the poststorage fermentation process, and therefore, dried CLP has the potential of being a natural preserving agent, especially in fermented products.

There was no significant difference between syneresis values (*p* > .05) among the treatments (Figure 1c). However, there was a decreasing nonsignificant trend of syneresis values as the inclusion levels of CLP increased. This could be attributed to the higher amounts of fiber that is included in T2, T3, and T4, as the ratio of CLP inclusion increased. The higher fiber content in dairy products may help to hold the water molecules inside the food matrix and thereby minimizing the syneresis. According to Ramulu and Rao. (2003), curry leaves contain approximately 16.3% (w/w) and 13.4% (w/w) of total dietary fiber and insoluble dietary fiber, respectively. Plant‐based fiber helps to reduce syneresis by holding the water molecules together when incorporated into fermented dairy products. Likewise, in a recent study (Staffolo et al., 2017) it was demonstrated that a semisolid dairy dessert made with plant‐based fiber exhibits low syneresis and low impaired mechanical properties as compared to dessert without added plant fiber.

### 3.2 Changes in fat, moisture, and total solid content of cream cheese

The addition of CLP did not significantly influence the fat, moisture, and total solid content of the samples (Table 1), indicating that CLP did not significantly influence the gross chemical composition of the cream cheese. This, perhaps CLP was added in minute proportion to the main ingredients and thus exerting a minimal effect on the gross composition. Yet, numerical values for fat content reduced with the addition of CLP (T2, T3, and T4) in contrast to the control was observed. However, the reasons behind this nonsignificant reduction of fat content are unknown, and therefore, further investigations are warranted in this regard. In a study by Das et al. (2011), it was discovered that using CLP can inhibit the formation of free fatty acids, lipid peroxide, and thiobarbituric acid reactive substances in meat. In this context, we speculated to lower the fat degradation (i.e., lipolysis) in cream cheese with increase CLP content, and therefore, incorporation of CLP will not affect gross fat content as opposed to the observation. Inhibition of free fatty acid formation in food products is beneficial to lowering the probability of rancidity and therefore off‐flavors. There was no significant difference in moisture content (Table 1). However, the moisture content of the samples T3 and T4 tends to be lower than the control (T1) sample. Moreover, there was no significant difference in total solids among the four treatments (Table 1), although there was an increase in the percentage of total solids in T2 (55.87%) and T4 (54.77%) than T1 (56.51%). This is likely because of added CLP into T2 and T4, which increases the dry mass of the samples.

**TABLE 1:** Changes in fat, moisture, and total solid content of cream cheese

Parameter	T1	T2	T3	T4
Fat (%)	45.59 ± 0.00	40.96 ± 0.00	40.82 ± 0.00	33.01 ± 0.00
Moisture (%)	56.51 ± 1.19	55.87 ± 0.06	54.62 ± 0.73	54.77 ± 1.08
Total solid content (%)	44.39 ± 1.98	46.22 ± 2.15	40.38 ± 4.28	45.37 ± 0.98

Note

Means with superscript asterisks marks are significantly different from other treatments, (*n* = 4; *p* < .05). T1, T2, T3, and T4 contain varying levels of dried CLP w/v = 0% (control), 0.15%, 0.2%, and 0.25%, respectively.

### 3.3 Microbiological analysis

Inclusion of CLP into cream cheese did not affect the microbiological quality (i.e., yeast and mold counts, coliform counts, and viable aerobic plate counts) in all studied treatments (T1‐4) on a considered storage time, indicating no influence on microbiota because of increasing concentration of CLP addition (Table 2). This is probably due to the effect of storage duration that allows the growth of respective microorganisms, as also indirectly indicated by increasing TA and decreasing pH. However, during the entire storage period, a gradual increase (*p* < .05) of yeast and mold count was observed (from 6.78 ± 0.00 CFU/ml to 7.48 ± 0.01 CFU/ml) for all cream cheese samples (T1‐4; Table 2). The highest viable yeast and mold counts were reported from the last day of storage (10th) compared with the lowest count on the day of production. At the end of the storage, T1, control (7.48 ± 0.01 CFU/ml) and T4 (7.32 ± 0.03 CFU/ml) tend to contain numerically higher and lower (nonsignificant) values of yeast and mold count, respectively (Table 2). This may be due to *Murraya koenigii* L. leaves and root methanolic extracts have shown to exhibit an antifungal effect on three kinds of fungal strains as reviewed by Heer et al. (2016). Likewise, at the end of the storage period, the viable coliform counts increased, indicating the highest and lowest values at the end of storage and beginning of storage, respectively. Upon storage, T1 and T4 tend to enumerate the nonsignificantly higher (2.51 ± 0.00 CFU/ml) and lower (2.40 ± 0.01 CFU/ml) viable coliform counts, respectively. The probable reason for the reduction of viable coliform counts in treatments with increased CLP inclusion could be due to the antibacterial effect of *Murraya koenigii* L. extracts. This is in agreement with Malwal and Sarin (2011), who showed growth inhibition of *E. coli* strain, from the hexane and methanolic root extracts of *Murraya koenigii* L., and thus exhibiting the antimicrobial activity of curry leaves extracts. Similarly, the viable aerobic counts increased during the storage, with nonsignificantly higher values in T2 (7.47 ± 0.01 CFU/ml) compared with T1 (7.20 ± 0.00 CFU/ml). In fact, we expected T1 would have resulted in higher values for the viable aerobic counts at the end of the storage, in accordance with the values recorded from the first and fifth days of storage. Nevertheless, we observed an abrupt decrease in viable aerobic counts in T1 from 6.26 ± 0.00 to 7.30 ± 0.00 CFU/ml, possibly due to a practical reason, as opposed to the expectation. Despite the nonsignificant lower values in control and reduction of the viable aerobic counts with increasing levels of CLP in the present study, the behavior agrees with Malwal and Sarin (2011), who showed the antibacterial effect of curry leaves.

**TABLE 2:** The variation in viable counts of yeast and mold, coliform, and viable aerobic plate counts in cream cheese samples during the storage period of 10 days (CFU/ml)

Treatment	Period of storage (Days)		
	1	5	10
Yeast and mold counts			
T1	6.85 ± 0.00^Aa^	7.02 ± 0.01^Ab^	7.48 ± 0.01^Ac^
T2	6.83 ± 0.00^Aa^	7.02 ± 0.00^Ab^	7.46 ± 0.00 ^Ac^
T3	6.81 ± 0.00^Aa^	7.01 ± 0.00^Ab^	7.38 ± 0.00^Ac^
T4	6.78 ± 0.00^Aa^	6.99 ± 0.01^Ab^	7.32 ± 0.03^Ac^
Coliform counts			
T1	1.63 ± 0.03^Ba^	1.70 ± 0.025^Bb^	2.51 ± 0.00^Bb^
T2	1.59 ± 0.01^Ba^	1.67 ± 0.00^Bb^	2.48 ± 0.00^Bc^
T3	1.52 ± 0.02^Ba^	1.70 ± 0.02^Bb^	2.44 ± 0.02^Bc^
T4	1.47 ± 0.00^Ba^	1.69 ± 0.00^Bb^	2.40 ± 0.01^Bc^
Viable aerobic plate count			
T1	6.26 ± 0.00^Ca^	6.47 ± 0.00^Cb^	7.30 ± 0.00^Cc^
T2	6.19 ± 0.02^Ca^	6.43 ± 0.01^Cb^	7.47 ± 0.01^Cc^
T3	6.17 ± 0.03^Ca^	6.42 ± 0.01^Cb^	7.42 ± 0.00^Cc^
T4	6.11 ± 0.00^Ca^	6.38 ± 0.00^Cb^	7.20 ± 0.00^Cc^

Note

Data are expressed as mean ± standard deviation; (*n* = 4); means in the same row without a common small letter significantly (*p* < .05) differ for each treatment; means in the same column without a common capital letter significantly (*p* < .05) differ among the treatments per microbial analysis; T1, T2, T3, and T4 contain varying levels of dried CLP w/v = 0% (control), 0.15%, 0.2%, and 0.25%, respectively.

### 3.4 Antioxidant analysis of cream cheese

All the CLP‐incorporated cream cheese samples (T2, T3, T4) showed higher (*p* < .05) amounts of TPC compared with the control (T1) cream cheese (Table 3). The results showed that the level of CLP incorporation greatly affected the TPC of cream cheese, that is, increasing TPC with increasing CLP content. T1 had the lowest TPC content (70.67 ± 1.16 mg GAE/100 g) while T4 had the highest TPC content (196.33 ± 4.73 mg GAE/100 g). This is because, the addition of dried CLP increases the TPC, since plants are in general rich in polyphenols. The TPC level observed in the control cream cheese may probably be derived from polyphenols in milk, which is mostly transferred from though animal feed, especially when the cows were provided with diets based on fresh forages (Besle et al., 2010; Di Trana et al., 2015). Another study (Vázquez et al., 2015) reported unpasteurized fresh cow milk generally contains TPC around 49.00 ± 10.77 mg GAE/L, which is lower than the values observed in the present study and the discrepancies in numerical values may relate to the botanical composition and amount of pasture given to animals in their study and our study. Similarly, increasing TPC has been observed with increasing levels of spinach powder incorporated cream cheese, in a recent study (El‐Sayed, 2020). Additionally, higher TPC values of cream cheese were reported for increasing levels of pomegranate peel extract by El‐Shafei et al. (2017). Since plants are rich sources of polyphenols and have higher concentrations of bioactive compounds, the incorporation of plant materials into cream cheese resulted in higher TPC.

**TABLE 3:** The antioxidant potentials of control and CLP‐incorporated cream cheese

Treatments	Total phenolic content (TPC) [mg GAE/100 g of sample]	DPPH assay (Average IC_50_ [mg/ml] value)	FRAP assay (Average mM Fe [II]/100 g)
T1	70.67 ± 1.16^E^	0.09 ± 0.00^A^	0.08 ± 0.01^D^
T2	114.33 ± 1.53^D^	0.08 ± 0.00^B^	0.13 ± 0.01^C^
T3	158.67 ± 1.53^C^	0.07 ± 0.0^C^	0.16 ± 0.06^B^
T4	196.33 ± 4.73^B^	0.06 ± 0.00^D^	0.17 ± 0.00^A^
CLP	1644.67 ± 4.73^A^	NA	NA

Note

Means with different superscripts are significantly different (*p* < .05); values are expressed as mean ± standard deviation; (*n* = 3).

Abbreviations: DPPH, 2,2‐diphenyl‐1‐picryl‐hydrazyl radical scavenging ability; FRAP, ferric reducing antioxidant power; NA, not available; T1, T2, T3, and T4 contain varying levels of dried CLP w/w = 0% (control), 0.15%, 0.2%, and 0.25%, respectively.

The results of the DPPH assay showed a gradual and significant increase in the antioxidant activity with each level of CLP inclusion (Table 3). The untreated sample (T1) had the highest IC_50_ value (0.09 ± 0.00) which indicates the lowest antioxidant capacity, while the T4 sample resulted in the lowest IC_50_ value (0.06 ± 0.00) and thus reflects the highest antioxidant capacity among the treatments. In curry leaves, the carbazole alkaloids (e.g., mahanimbine and koenigines) have been reported to exhibit antioxidant potentials (Ramsewak et al., 1999; Tachibana et al., 2001; Tachibana et al., 2003). Hence, it can be assumed that the higher antioxidant capacity observed in the CLP‐incorporated cream cheeses is a direct impact of carbazole alkaloids in CLP that was added into the cream cheese base in the current study. Similar observations were made when curry leaves essential oil incorporated with a milk‐based confection, *burfi*, a popular confection of the Indian sub‐continent (Badola et al., 2018). Authors reported the inclusion of curry leaves essential oil into *burfi* enhanced the storage stability without compromising the sensory acceptability.

Similarly, in the FRAP assay, a gradual increase in the total antioxidant capacity, with increased CLP content, was observed among all the samples. The T1 (0% CLP) sample recorded the lowest level (0.83 ± 0.01 mM Fe (II)/100 g) of antioxidant capacity as indicated from FRAP, whereas the highest amount was recorded from the T4 (0.17 ± 0.00 mM Fe [II]/100 g). This is also because *Murraya koenigii* L. possessing antioxidant capacities originate from its bioactive secondary metabolite, that is, mahanimbine, which is the most common bioactive compound based on FRAP assay (Ganesan et al., 2013; Ng et al., 2018). Likewise, a recent study (Ghasemzadeh et al., 2014) also revealed that methanol extracts of curry leaves contain an average FRAP value of 0.644 mM of Fe (II)/g. The differences in FRAP values might be an effect of differences in concentration of curry leaves in samples in their study (e.g., leaf extracts) and our study (e.g., cream cheese). Furthermore, the authors reviewed curry leaves have been reported to have antitumor, antioxidant, anti‐inflammatory, and antihyperglycemic effects. Thus, incorporation of dried CLP into cream cheese in our study might confer such health effects, and therefore, those potentials need to be further investigated.

### 3.5 Sensory evaluation

**FIGURE 2:**
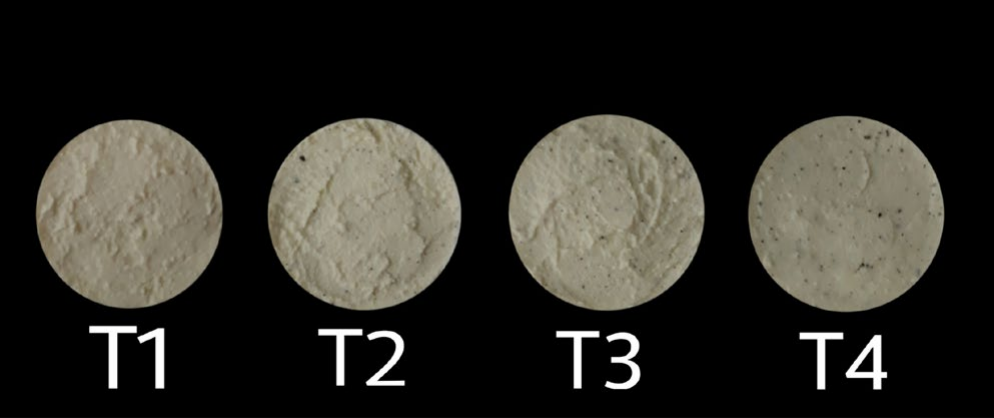
Dried curry leaves powder (CLP)‐incorporated fresh cream cheeses. T1, T2, T3, and T4 contain varying levels of dried CLP w/v = 0% (control), 0.15%, 0.2%, and 0.25%, respectively

**FIGURE 3:**
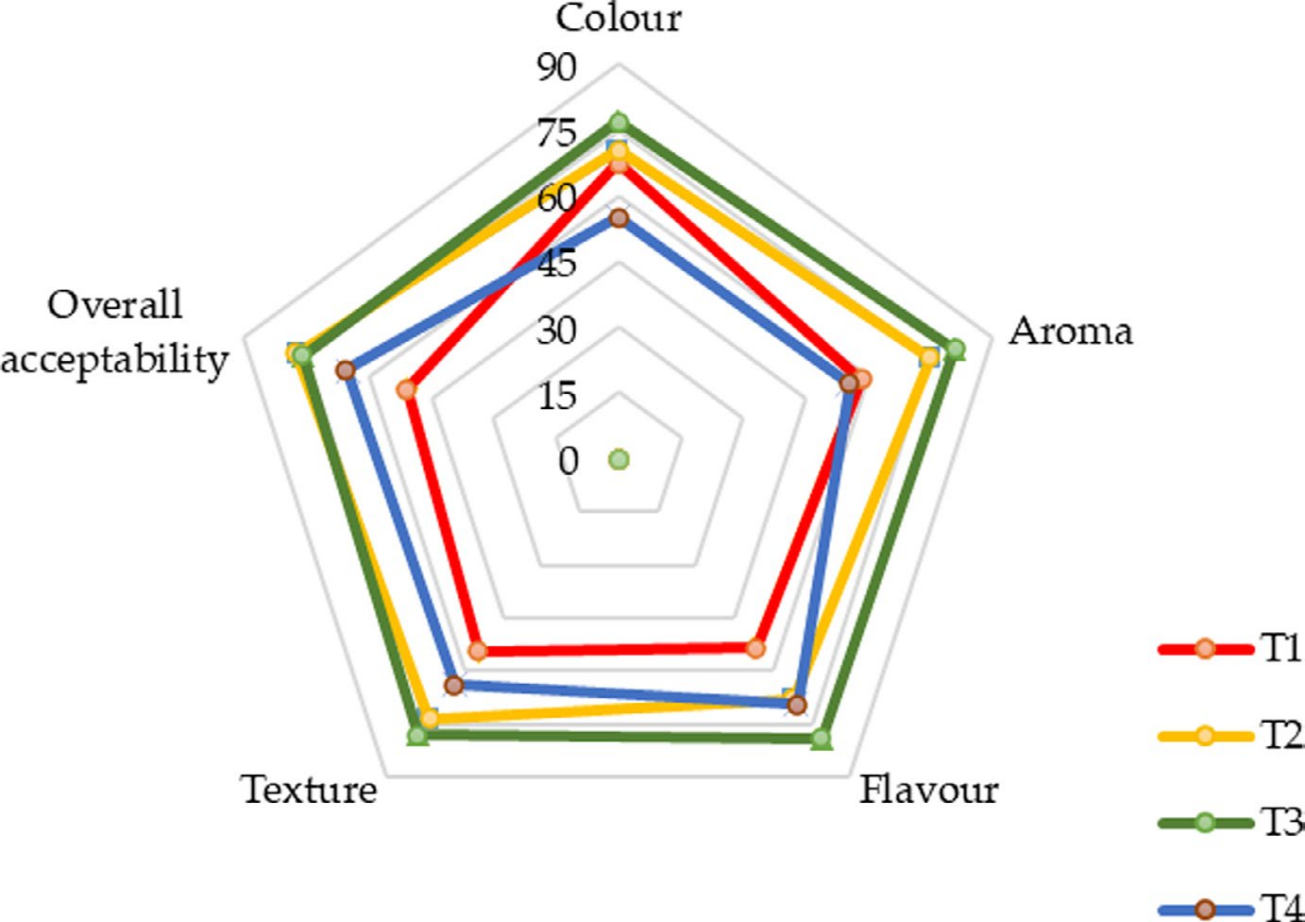
Variation of sensory properties in cream cheese with varying levels of CLP incorporations. T1, T2, T3, and T4 contain varying levels of dried CLP w/v = 0% (control), 0.15%, 0.2%, and 0.25%, respectively

The appearance of CLP‐incorporated cream cheese is shown in Figure 2. The sensory evaluation revealed that the 0.2% CLP (T3) ranked the highest score for color, aroma, flavor, texture, and overall acceptability while the 0% CLP (control) ranked the lowest acceptance for tested organoleptic properties (Figure 3), thus demonstrating the possibilities of developing a cream cheese containing 0.2% of CLP with an acceptable level of organoleptic properties. α‐pinene, sabinene, ẞ‐pinene, 1‐phenylethanethiol, linalool‐like odorants with flavor dilution (FD) factors majorly contribute to the aroma of curry leaves (Verma et al., 2012). The unique sulfur and burnt odor is a result of 1‐phenylethanethiol in combination with its high FD factor (Steinhaus, 2017). Its ability to produce a strong aroma and flavor is beneficial in terms of using CLP as a condiment in many food applications and preparations. The study by Shanthala and Prakash (2005) assessed the acceptability and attitude toward the consumption of curry leaves‐incorporated products (e.g., bread, seasoned potatoes, and cooked rice) and found 22% of panelists would increase the consumption of CLP‐incorporated food products due to the associated health claims, whereas 20% considered its taste to be acceptable, and 58% responded their decision was influenced by both health benefits and taste. Thus, the incorporation of CLP into cream cheese in our study also likely to be positively influenced the acceptance by prospective consumers. However, the level of CLP incorporation is critically important, since smaller amounts of dried CLP were more acceptable to the majority of the panelists in their study (Shanthala & Prakash, 2005). Accordingly, the results of the current study also show that the aroma and the flavor are strongly influenced by the level of incorporation and thus the moderate level of CLP included cream cheeses (0.2%; T3) resulted in the highest scores for the studied sensory attributes. Hence, the addition of CLP should be done carefully to preserve the organoleptic quality attributes with a delicate balance.

### 3.6 Principal component analysis

**FIGURE 4:**
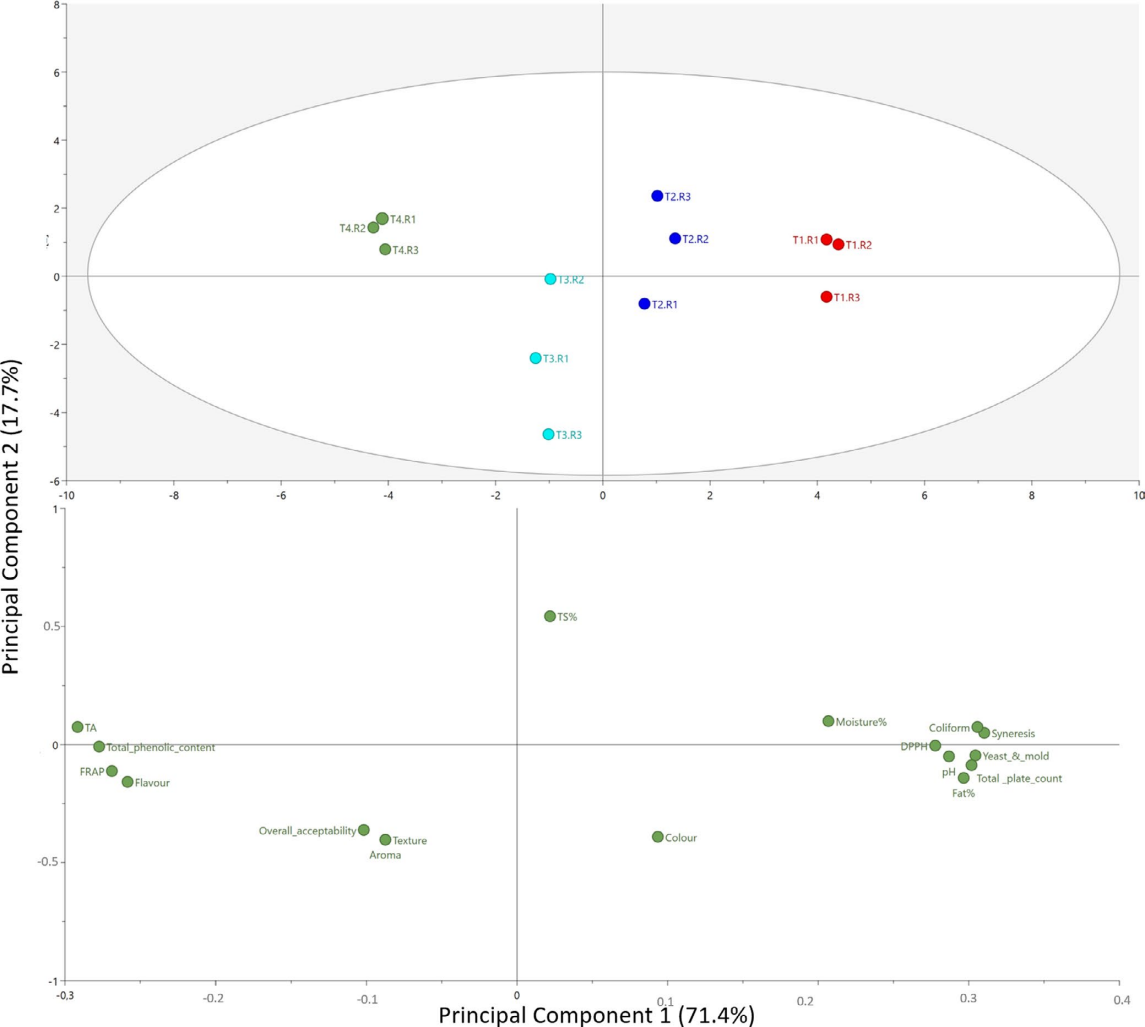
Principal component analysis (PCA) score plot (upper panel) and loading plot (lower panel) of all studied parameters and their relationship with T1, T2, T3, and T4 containing varying levels of dried CLP w/w = 0% (control), 0.15%, 0.2%, and 0.25%, respectively

A PCA model, explaining 71.4 and 17.7 percent of the variance in the first two principal components, respectively, was used to evaluate the overall variation of studied parameters. The score plot (Figure 4, upper panel) suggested that CLP infused cream cheeses were different from each other as well as from the control sample. T1 and T2 are located on the same side (right) of the PCA, while T3 and T4, which containing a comparatively higher amount, located on the opposite side (left) of the score plot. According to the loading plot (Figure 4, lower panel), the underlying reasons for this variation could be comprehended. T1 (control sample) showed comparatively greater influence from moisture%, fat%, total solids%, syneresis, pH, DPPH as well as microbiological quality (i.e., higher viable aerobic plate counts, coliform counts, yeast, and mold counts). T2 showed moderate influence from all observed variations. T3 had a higher influence from sensory parameters (i.e., aroma, texture, and overall acceptability) compared with other treatments. T4 (0.25% CLP) had higher titratable acidity, total phenolic content, FRAP, and flavor. According to the loading plot, we could observe the cream cheese samples with higher FRAP resulted in higher titratable acidity and total phenolic content, while lower DPPH and microbiological contents. Total phenolic content was higher in those samples, which resulted in greater levels of flavor scores and lower levels of color scores from the sensory analysis. This probably resulted from the high amount of curry leaf flavanols including myricetin‐3‐galactoside, quercetin‐O‐pentohexoside, quercetin‐3‐diglucoside, quercetin‐3‐O‐rutinoside, quercetin‐3‐glucoside, quercetin‐3‐acetylhexoside, and the curry leaf phenolics that prevent compound peroxidation (Singh et al., 2011).

Also, it showed when moisture percentage is higher, microbiological counts were also elevated. This could be explained by the fact that food with a higher water activity (a_w_) provide a suitable substrate for microbial growth. Generally, in fresh cream cheeses, the a_w_ is as higher as 0.991% and the moisture percentage (wet basis) is around 45%–53% (Schmidt & Fontana, 2008). Moreover, higher syneresis was observed when there is more moisture in the samples, which contain a higher amount of fat as well. This phenomenon was highlighted in the control (0%) and the T1 (0.15%) CLP containing samples.

Further work will merit the understanding of incorporating CLP into dairy products. For this, characterizing the phenolic profile of CLP using high‐performance liquid chromatography (HPLC) and liquid chromatography–mass spectrometry (LC‐MS) would be beneficial. Innovative and emerging processing technologies for the processing of curry leaves and cream cheese production should be tested to enhance or preserve the natural antioxidant capacities in curry leaves. The specific nutritional, therapeutic, and nutraceutical potentials of bioactive compounds in CLP‐incorporated cream cheese should be further researched since those aspects were beyond the scope of the current study.

## 4 CONCLUSIONS

Dried curry leaves (*Murraya koenigii* L.) could be successfully incorporated into fresh cream cheese without compromising its physicochemical properties, while the addition of curry leaves powder significantly increased the antioxidant activity of the cream cheese. The inclusion of CLP slowed the pH reduction and hindered the syneresis during the 10‐day storage period. The microbial quality of the CLP‐incorporated cream cheese was better compared with the control sample with an exception in the viable aerobic count. Sensory evaluation results confirmed that the 0.2% inclusion level of CLP had the highest acceptance for organoleptic properties such as color, aroma, and texture. PCA enabled to distinguish the variation of CLP infused cream cheeses, as influenced by the overall variation of physicochemical, sensory, antioxidant, and microbiological properties. PCA further enabled to study of the relationship between studied parameters. Further research, however, is needed to evaluate the functional properties of dried curry leaves‐incorporated cream cheeses. Thus, the experimental results of this study offer a potential scope for new product developments for the industry as well as new insights into future research.

## CONFLICT OF INTEREST

The authors declare no conflict of interest.

## PERMISSION TO REPRODUCE MATERIAL FROM OTHER SOURCES

Contain only originals.

## Data Availability

The data that support the findings of this study are available from the corresponding author upon reasonable request.
